# Three-Dimensional Spatial Analyses of Cholinergic Neuronal Distributions Across The Mouse Septum, Nucleus Basalis, Globus Pallidus, Nucleus Accumbens, and Caudate-Putamen

**DOI:** 10.1007/s12021-022-09588-1

**Published:** 2022-07-06

**Authors:** Andres Carrasco, Dorothy E. Oorschot, Paolo Barzaghi, Jeffery R. Wickens

**Affiliations:** 1 Neurobiology Research Unit, Okinawa Institute of Science and Technology Graduate University, Okinawa, Japan; 2 Department of Anatomy, School of Biomedical Sciences, and the Brain Health Research Centre, University of Otago, Dunedin, New Zealand; 3 Imaging Section, Okinawa Institute of Science and Technology Graduate University, Okinawa, Japan

**Keywords:** Acetylcholine, Axonal bundles, Cognition, Forebrain, Topography

## Abstract

Neuronal networks are regulated by three-dimensional spatial and structural properties. Despite robust evidence of functional implications in the modulation of cognition, little is known about the three-dimensional internal organization of cholinergic networks in the forebrain. Cholinergic networks in the forebrain primarily occur in subcortical nuclei, specifically the septum, nucleus basalis, globus pallidus, nucleus accumbens, and the caudate-putamen. Therefore, the present investigation analyzed the three-dimensional spatial organization of 14,000 cholinergic neurons that expressed choline acetyltransferase (ChAT) in these subcortical nuclei of the mouse forebrain. Point process theory and graph signal processing techniques identified three topological principles of organization. First, cholinergic interneuronal distance is not uniform across brain regions. Specifically, in the septum, globus pallidus, nucleus accumbens, and the caudate-putamen, the cholinergic neurons were clustered compared with a uniform random distribution. In contrast, in the nucleus basalis, the cholinergic neurons had a spatial distribution of greater regularity than a uniform random distribution. Second, a quarter of the caudate-putamen is composed of axonal bundles, yet the spatial distribution of cholinergic neurons remained clustered when axonal bundles were accounted for. However, comparison with an inhomogeneous Poisson distribution showed that the nucleus basalis and caudate-putamen findings could be explained by density gradients in those structures. Third, the number of cholinergic neurons varies as a function of the volume of a specific brain region but cell body volume is constant across regions. The results of the present investigation provide topographic descriptions of cholinergic somata distribution and axonal conduits, and demonstrate spatial differences in cognitive control networks. The study provides a comprehensive digital database of the total population of ChAT-positive neurons in the reported structures, with the x,y,z coordinates of each neuron at micrometer resolution. This information is important for future digital cellular atlases and computational models of the forebrain cholinergic system enabling models based on actual spatial geometry.

**Supplementary Information:**

The online version contains supplementary material available at 10.1007/s12021-022-09588-1.

## Introduction

Cholinergic neurons in the central nervous system modulate attention (Ljubojevic et al., 2018; Schmitz & Duncan, 2018), motivation (Dulawa & Janowsky, 2019), learning (Aoki et al., 2015; Conner et al., 2003; Deiana et al., 2011), and memory (Drachman & Leavitt, 1974; Easton et al., 2020). Cholinergic somata in the forebrain are predominantly located in subcortical nuclei and are characterized by local and distal networks of communication (Mesulam et al., 1984). Developments in transgenic mouse lines with cell specific expression of fluorescence or conditional expression of fluorescent proteins (such as the ChAT-IRES-Cre mouse used in the current study), together with the availability of high volume computer storage capacity, image processing software, and high performance workstations, have enabled brain-wide 3-D visualization and localization of entire neurochemically defined populations (Li et al., 2018; Matamales et al., 2016). These developments also allow the locations of neurons to be expressed in a Cartesian coordinate system. This permits application of mathematical tools for quantifying spatial relationships, thereby extending our understanding of the architectural principles associated with cognitive regulation. The present study used this approach to examine spatial relationships of cholinergic neurons within the following subcortical nuclei: septum (S), nucleus basalis (NB), globus pallidus (GP), nucleus accumbens (NA), and caudate-putamen (CPu).

The spatial relationships of cholinergic neurons in these networks may be important for their distinct functions. For example, cortical cholinergic innervation originates in a compact group of projection neurons in the NB, whose somata are distant from the projection area (Nemy et al., 2020; Turchi et al., 2018). On the other hand, the striatal cholinergic innervation originates largely from an intrinsic population of cholinergic interneurons whose axons ramify extensively in the space around the cholinergic interneuron somata indicating local regulation (Abudukeyoumu et al., 2019; Bolam et al., 1984; Kimura et al., 1981). In addition, the presence of axon bundles in the CPu imposes spatial restrictions in cholinergic innervation patterns distinct from the architectural constraints of NB networks (Mesulam et al., 1983; Tepper & Koos, 2017). Based on these observations, we hypothesized that the spatial organization of cholinergic cells in subcortical nuclei are heterogeneous and are modulated by properties of efferent distributions and axonal conduits.

Large data sets of high resolution images acquired from transgenically and immunohistochemically stained cholinergic neurons now enable areal and volumetric measures of cholinergic distributions to be obtained and compared with theoretical distributions under various constraints. Methods based on random point process theory frameworks and graph signal processing techniques provide new measures that can be applied to cell body arrangements (Bolanos et al., 2013; Jacobsen, 2006). Here we apply such spatial analysis methods to an exhaustive three-dimensional map of the location of cholinergic cell somata to establish spatial and topological descriptions of subcortical cholinergic nuclei. The total population of cholinergic cells in selected forebrain nuclei of a single mouse brain were analyzed. The results provide a comprehensive description of the cell body distributions of cholinergic and axonal networks and identify spatial arrangements of subcortical cognitive modulator nuclei in a mouse forebrain, which provides new information relevant to mice of the same strain. The study provides a comprehensive digital database of the total population of ChAT-positive neurons in the reported structures, with the x, y, z coordinates of each neuron, together with the somal size, at micrometer resolution. This complements previous descriptions of the basal forebrain cholinergic projection neurons (Nadasdy et al., 2010) by adding a comparison with cholinergic interneurons in the CPu and NA, and statistical spatial analysis. This information is important for future models of the brain cholinergic system enabling models based on actual spatial geometry.

## Experimental Methods

The forebrain of an 8-week-old female mouse was used to characterize morphological and spatial features of cholinergic neurons across the subcortical nuclei. The animal was the product of breeding of ChAT-IRES-Cre and 129S-Gt(ROSA)26Sortm32(CAG-COP4*H134R/EYFP)Hze/J mice (Stock#: 012,569 Jackson Laboratories). This breeding yielded a mouse with ChAT-positive cholinergic neurons that expressed green fluorescence. Animal housing followed standard laboratory conditions on a reversed 12/12 h light/dark cycle with 5 female animals per cage. Experimental procedures were approved by the Committee for Care and Use of Animals at the Okinawa Institute of Science and Technology Graduate University.

The distribution of cholinergic neurons in the anterior subcortical nuclei of the forebrain permitted efficient automatic and reliable identification of neuronal locations. In contrast the dense arrangement of cholinergic neurons in various regions of the brainstem inflicted unwanted noise levels to the thresholding algorithms employed. Therefore, the present investigation was limited to the regions that could adequately and efficiently be quantified. For brainstem nuclei a different approach would be required.

### Perfusion, Immunocytochemistry, and Microscopy

A deeply anesthetized (5% isoflurane) mouse was perfused (~ 0.75 mL/min) with saline and 4% paraformaldehyde in a phosphate buffer solution (PBS, pH 7.4, Thermo-Fisher, 10010) through the ascending aorta. The brain was removed, submerged in 30% sucrose, and embedded in gelatin (Liu et al., 2017). The sucrose treatment was part of a standard protocol used in our laboratory on the assumption that it allows the tissue to remain more pliable (Gibb & Kolb, 1998). A vibrating microtome (VT1000S, Leica Microsystems) was employed to cut 60 μm sections in the coronal plane from the most anterior part to the most posterior part of the CPu. Slices were rinsed three times with PBS at room temperature and permeabilized for 45 min with a mixture of 0.1% bovine serum albumin (BSA, Sigma), 5% normal donkey serum (NDS, Chemicon), and 0.3% triton X-100 (Bio-Rad) in PBS. Three more rinses with PBS were conducted prior to overnight incubation with primary antibodies: goat anti-ChAT (Millipore, AB144P, 1:150) and chicken anti-YFP (abcam, ab13970, 1:500) in triton-free blocking solution (5% NDS, 0.1% BSA and PBS) at 4 °C. The YFP antibody was used to increase the intensity of the intrinsic YFP fluorescence signal to make it comparable to the intensity of the ChAT signal. The following day, slices were rinsed and stained with secondary antibodies for 2 h: Alexa Fluor 594 donkey anti-goat (Invitrogen, A-11058, 1:500) and Alexa Fluor 488 donkey anti-chicken (Jackson ImmunoResearch, 703–545-155, 1:500) to yield magenta (ChAT) and green (YFP) fluorescence. Sections were rinsed three more times with PBS solution and mounted on glass slides. Image acquisition was conducted on an inverted confocal microscope (Zeiss LSM 880) at 20x. Photomultiplier gain, pinhole size, and laser power were set and kept constant for all subsequent image acquisitions. Alexa 488 was excited at 488 nm and detected at 510–580 nm and Alexa 594 was excited at 561 nm and detected at 585–735 nm. Step sizes of 1.4 μm (z-stack) and pixel sizes of 0.425 μm in X and Y directions were achieved. Images were stitched using Imaris 9.0 (Bitplane). Co-localization of magenta and green fluorescence was examined.

### Delineation of Regional Boundaries

Since the sections were stained for ChAT and YFP, the delineation of regional boundaries was made with reference to internal white matter structures that could be identified, the density of the cholinergic neurons, and the atlas of Franklin and Paxinos (2008). We have not used the coordinates from the atlas because it was based on a larger male brain and the brain used in the present study was a smaller female brain.

The CPu was delineated by white matter structures superiorly and laterally (subcortical white matter), by the lateral ventricle medially, and inferiorly by a line drawn between the anterior commissure and the inferior limit of the subcortical white matter. Furthermore, in the anterior part of the CPu the external capsule and claustrum (laterally), forceps minor of the corpus callosum (medial and dorsal) and anterior commissure anterior part (ventrally) were used as borders. In the posterior part of the CPu the cerebral cortex and external capsule (laterally), the lateral ventricle (medially) the external capsule (dorsally), GP and anterior commissure posterior part (ventrally) were employed as landmarks of CPu borders.

When the GP appeared in more posterior sections it was delineated from the CPu by its lower density of cholinergic neurons and increased density of white matter. This lower density of cholinergic neurons was limited by the CPu (laterally and dorsally), internal capsule (medially), and separated from the ventral pallidum by a line drawn medially from the posterior part of the anterior commissure.

In more posterior sections containing the GP, the NB was delineated medially by the increased density of cholinergic neurons relative to the GP and CPu. It was initially located at the inferior end of the internal capsule, and then was located inferio-medially to the GP and inferiorly within the internal capsule. The NB extended as clusters of cells along the inferior boundary of the GP and CPu.

The S was identified in the medial regions of the sections and inferior to the corpus callosum (Franklin and Paxinos (2008)). Lateral borders were delimited by the right and left lateral ventricle. The median preoptic nucleus served as a ventral boundary.

The NA was delimited by the CPu (laterally), lateral septal nucleus (medially), CPu (dorsally), and ventral pallidum (ventrally).

### Identification of Cholinergic Neurons

**Fig. 1:**
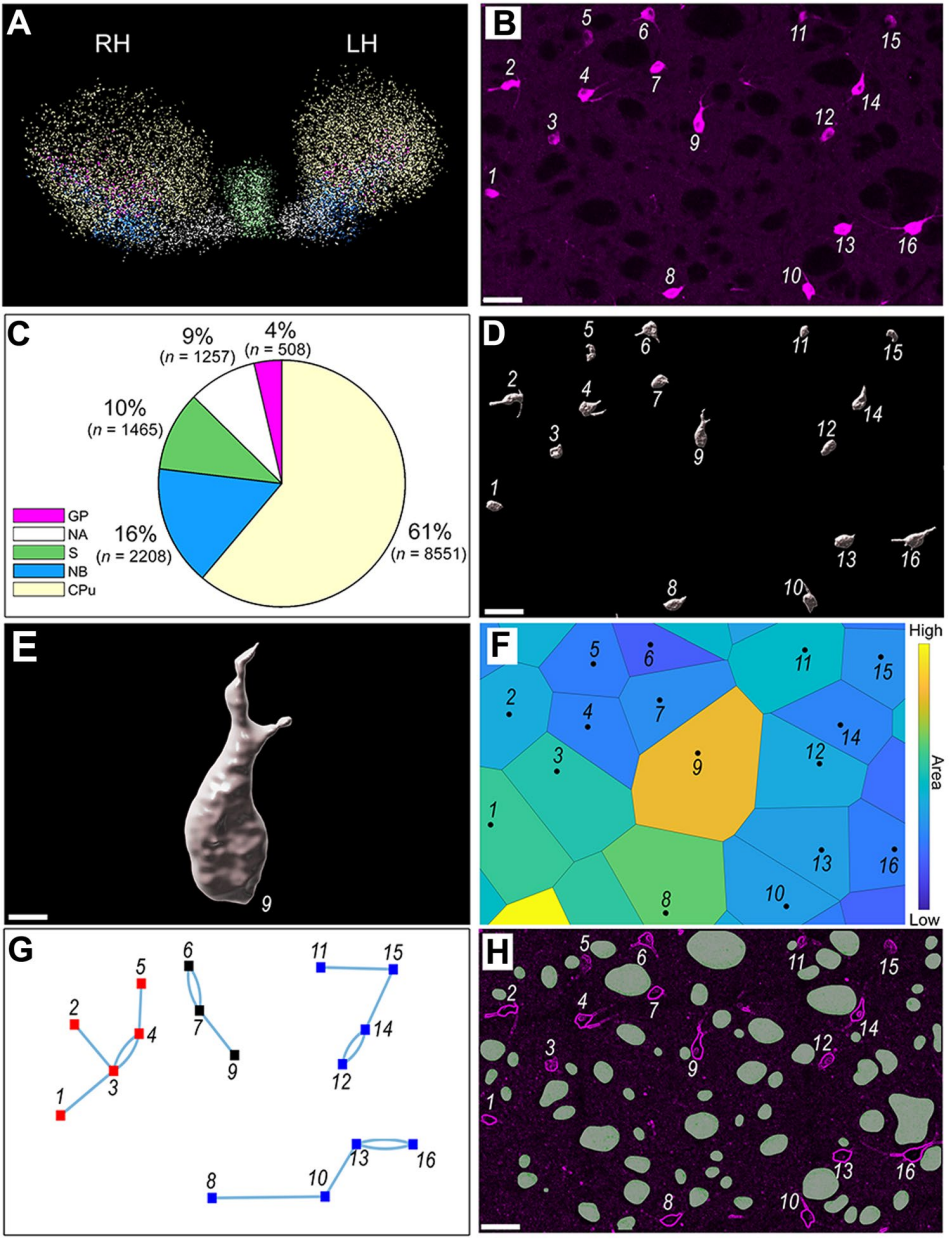
Experimental and analytical methods. **A** Color-coded spatial distribution of cholinergic neurons. Delineation of area boundaries followed the mouse brain atlas by Franklin & Paxinos, 2008. Color codes: light yellow: CPu, blue: NB, purple: GP, green: S, white: NA. RH: Right hemisphere, LH: Left hemisphere. *See Supplementary animation*. **B** Representative example of expression of ChAT in the CPu. Scale bar 50 μm. **C** Proportion and absolute number of cholinergic neurons across the bilateral CPu, NB, GP, S, and NA. **D** Volumetric representation of neurons identified in panel B. **E** High resolution image of neuron number 9 presented in panel D. **F** Voronoi tessellations of neuronal locations presented in panel B. Individual tessellations are color-coded by the size of the polygons. **G** Nearest neighbor analysis of neuronal locations presented in panel B. **H** Representative tracing of the axonal bundles (filled shapes) of the micro-photograph illustrated in panel B. Abbreviations: CPu: caudate-putamen; NB: nucleus basalis

To automatically identify and determine coordinates for cholinergic neurons, a threshold algorithm based on the fluorescence intensity and size of the soma was used. The ChAT signal was intense and easily separated from the background (Fig. 1B, Supplementary Fig. 1). To avoid counting non-somatic expression, a size threshold of 15 µm in diameter was set using Imaris 9.0 inbuilt algorithms. The centroid of the identified neurons was then encoded into 3D coordinates which were stored on a computer for further analysis.

### Data Analyses and Statistics

Identification of cholinergic neurons was from medial to lateral and through the depth of each section over all 59 coronal sections using commercial (Imaris 9.0, Bitplane) and in-house programs (Matlab). The anterior to posterior extent of each entire structure (CPu, GP, NA, NB and S) was exhaustively examined within this set of sections. Three-dimensional maps were generated by aligning anatomical landmarks across sequential images and spatial analyses and somal diameter measures were conducted. For the CPu, GP, NA, and NB, the right and left side was mapped.

To compute Voronoi tessellations, the three-dimensional space around the coordinates of cholinergic neurons was parcellated into segregated regions (R(i)) using Delaunay triangulation (Boundary function, Matlab). This procedure guaranteed that any point within the region R(i) was closer to the coordinates of the cholinergic neuron soma in question C(i) than to any other cholinergic neuron in the population. The volume of these regions was measured and plotted to show the inter-neuronal space between cholinergic neurons. The total volume and shape of each of the five regions examined was calculated by using a generalization of the convex hull and a subgraph of the Delaunay triangulation (Matlab) using the coordinates of the cholinergic neuronal somata.

Distances between cells were computed and nearest neighbor undirected graphs were constructed. To compute the nearest neighbor, the Euclidean distance between every pair of somata was calculated and the shorted distance found by exhaustive pairwise comparison.

Cell bodies (nodes) in connected clusters were measured and color-coded maps of cluster size were generated. Axonal bundles in the CPu were manually traced across sections and collapsed in a two-dimensional antero-posterior heat map. Last, measures of the clustering and regularity of cell distributions were performed by computing Ripley’s K-functions for experimental neuronal locations as well as for 1,000 simulations of random distributions. Ripley’s K-function (Baddeley et al., 2014; Ripley, 1977) provides a quantitative measure of the structure of a point pattern, and has been widely used in the analysis of point patterns in many fields including neuroanatomy (Anton-Sanchez et al., 2014; Jafari-Mamaghani et al., 2010; Jammalamadaka et al., 2013; Larsen et al., 2021; Matamales et al., 2016). To calculate Ripley’s K function we used in-house programs (Pointcloud, Matlab) to determine the number of neurons (K) within a given radius (r) of each other, using the formula:

$$K\left(r\right)=|V|\frac{{\sum }_{i=1}^{n}{\sum }_{j\ne i}I[D\left(i,j\right)\le r]}{{n}^{2}}$$

where V is the volume within radius *r*, *n* is the number of neurons in the population, *I* $[D\left(i,j\right)\le r]$ is the indicator function which is 1 if the distance from point (i) to point (j) is less than or equal to radius r (Ripley, 1981). This formulation is uncorrected for edge effects. The edge effects were not corrected because we compared the observed distribution with simulated, completely spatially random, distributions with the same shape and hence the same edges. In this analysis the uncorrected measure of the simulated distribution was biased in the same way as the experimental data set. According to Baddeley, 1993, “Edge effects can largely be ignored in hypothesis testing (Baddeley et al., 1993). In the standard Monte Carlo test of a simple null hypothesis, one uses an uncorrected (hence biased) version of a summary statistic, simulates a number of realizations of the null hypothesis and ranks the results according to some one-dimensional criterion. See (Diggle & Gratton, 1984; Diggle et al., 1991; Hall, 1988; König et al., 1991; Ripley, 1981, 1988)”. Statistical comparisons between experimental and simulated K-functions were conducted with paired sample t-tests.

To test for homogeneity in the density of cholinergic neurons, we defined pairs of sub-regions in each structure. We then tested whether the point densities were equal, based on the observed numbers of points (n_1_) and (n_2_) in sub-regions with measures v(W_1_) and v(W_2_) using the following formula (Illian et al., 2008) to calculate the F statistic:

$$F=\frac{v({W}_{1})(2{n}_{2}+1)}{v(2)(2{n}_{1}+1)}$$

When there was a significant departure from homogeneity in the samples tested (in the CPu and NB, see 7), we used an extended version of Ripley’s *K* function for non-constant intensity spatial point processes (Baddeley et al., 2001). The inhomogeneous *K* function was obtained using the following equation (Marcon & Puech, 2009) where ${\lambda }_{i}$ and ${\lambda }_{j}$ are the local densities of the points i and j, and as before *I* $[D\left(i,j\right)\le r]$ is the indicator function which is 1 if the distance from point (i) to point (j) is less than or equal to radius r:

$$\mathrm{K}inhom(r)=\frac{{\sum }_{i=1}^{n}{\sum }_{j\ne i}I[D\left(i,j\right)\le r]}{{{\lambda }_{i}\lambda }_{j}}$$

To calculate K_inhom_(r) we used in-house programs (Mathematica V3, Wolfram). To overcome the negative bias in intensity estimates at the boundary of the CPu (Burguet & Andrey, 2020) we extracted a sub-region that was separated from the boundary and estimated the intensity from the total region. A kernel estimation (Silverman, 1986) was used for ${\lambda }_{i}$ and ${\lambda }_{j}$ and was calculated using the Mathematica function “SmoothPointDensity” with method “SmoothKernel”. The standard Monte Carlo test was used, with 99 realizations of the null hypothesis, which was based on the inhomogeneous Poisson process using the local densities calculated from the observed sample. Edge correction was not undertaken on the basis that observed and simulated distributions had the same 3D shape and the K-function would therefore be similarly biased (Baddeley et al., 1993, 2014). Sampling a subregion was not necessary for the NB because of its gradually decreasing density approaching the boundary, causing less edge effect.

## Results

Both ChAT and YFP immunoreactivity reliably detected cholinergic neurons (Fig. 1A, B, Supplementary animation, Supplementary Fig. 1), and each method provided different advantages. ChAT immunoreactivity was particularly useful for the identification of cholinergic somata. YFP immunoreactivity provided complementary information by labelling the dendritic arborization and, by its absence, clearly outlining the axonal bundles in the CPu. For the analyses of the 3D spatial distributions, cholinergic neurons were immunohistochemically identified by their expression of ChAT (Fig. 1B) in 59 serial and sequential coronal sections. A custom segmentation algorithm was used to identify cells and reconstruct the shape of the cell body. Each cholinergic cell body was then plotted in three dimensional space and the entire population rendered to visualize their individual shapes and spatial distribution (Fig. 1A, Supplementary animation). Some asymmetry in the total number and density of cholinergic neurons is evident for the right versus left side, and this is quantified in Table 1. Overall, across the structures examined, there are 557 (3.98% of the total) more cholinergic neurons in the left hemisphere, though the asymmetry varies between structures. The overall density across the structures examined is 303.80 mm^−3^ in the left hemisphere (volume 23.94 mm^3^) and 277.64 mm^−3^ in the right (volume 24.19 mm^3^) a difference in density of 26.16 mm^−3^. Thus the structures examined in the left hemisphere have a higher number of cells that are more densely packed into a slightly smaller volume than the right.

The spatial distribution of cholinergic neurons was determined from the three-dimensional coordinates of the centroid of the cell body. The coordinate system employed placed X = 0 (right side, most lateral), Y = 0 (most anterior), and Z = 0 (most dorsal), and all coordinates were in relation to this reference point, in micrometers. Using the appropriate transformation to align this point to a reference atlas should allow the display of the dataset in applications for rendering three-dimensional neuroanatomical data. For the whole brain, a total of *n* = 13,989 cholinergic neurons were identified and registered within the regions of interest (NA, CPu, S, NB, GP) based on a mouse brain atlas (Franklin & Paxinos, 2008). The number in each region and proportion of the total are shown in Fig. 1C. The total number of neurons in each region of interest, bilaterally, were CPu (*n* = 8,551; 61%), NB (*n* = 2,208; 16%), S (*n* = 1,465; 10%), NA (*n* = 1,257; 9%), and GP (*n* = 508, 4%). The analysis of cell shape revealed that cholinergic neurons have comparable diameters irrespective of location (CPu: 25.32 ± 0.07 μm, NB: 26.54 ± 0.16 μm, S: 23.60 ± 0.17 μm, NA: 24.33 ± 0.22 μm, GP: 27.37 ± 0.34 μm, mean ± SD).

For the analysis of spatial distribution, each cell was assigned an identifying number (Fig. 1D), and the coordinates of the centroid of each identified cell body were registered in the database. The three-dimensional morphology of each cell's surface (Fig. 1E) was recorded. The Voronoi tessellations, for illustrative purposes shown in two dimensions (Fig. 1F), were also determined. Neighborhood relationships were assessed based on intercentroid (i.e. intersomal) three-dimensional Euclidean distances, illustrated in two dimensions (Fig. 1G).

The CPu is traversed by bundles of axons that provide pathways of communication between distant neurons. These highly dense regions of axonal fibers do not contain cholinergic neuronal somata. Hence, the presence of numerous fiber bundles in the CPu constrains the location of cholinergic neurons in this region (Fig. 1H) and could influence their spatial distribution.

### Axonal Bundles in the CPu

**Fig. 2:**
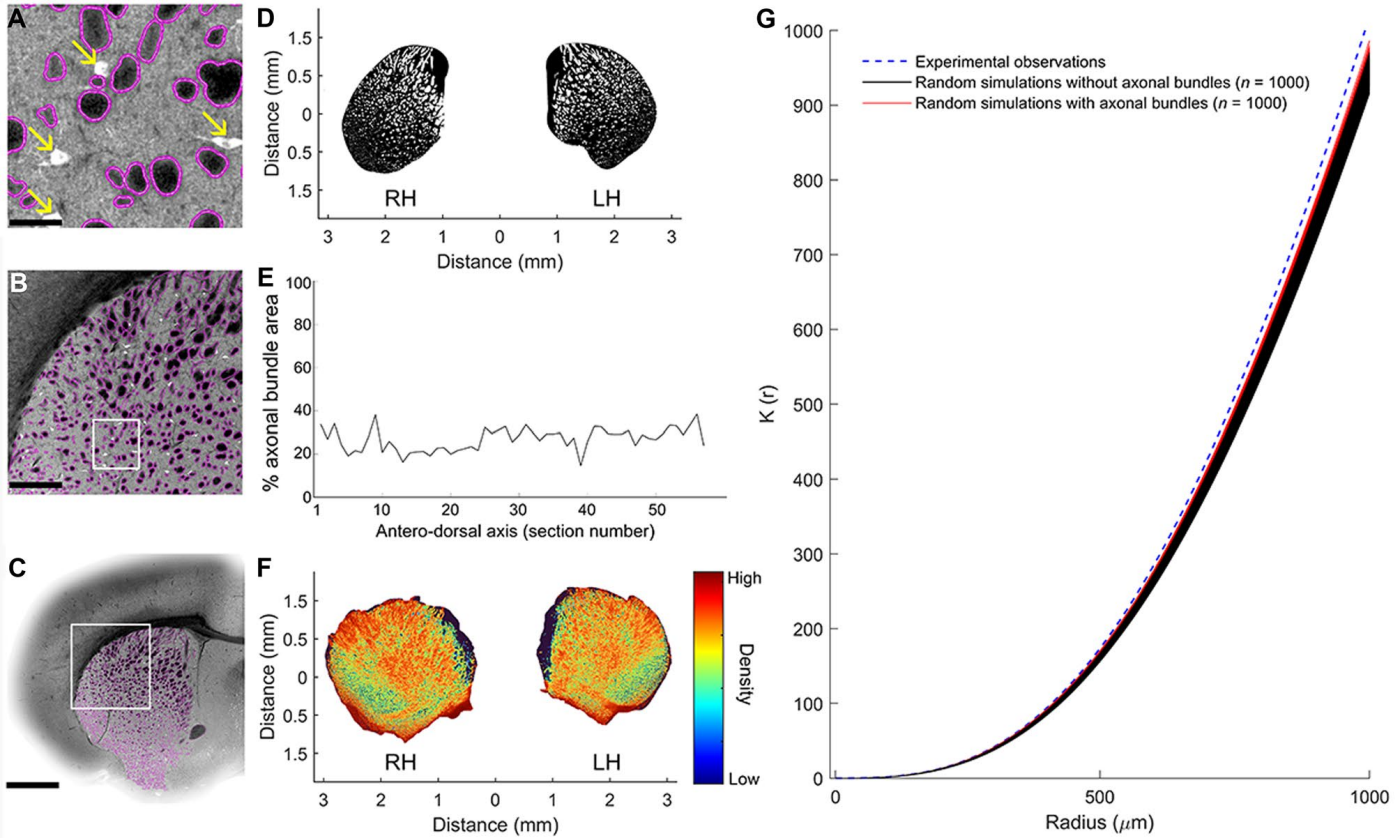
Effects of axonal fiber bundles in the CPu. **A**-**C** Representative analyses of axonal bundle tracing in the CPu at three levels of magnification (scale bars, A: 50 μm, **B**: 500 μm, C: 1,000 μm). Notice in A that the position of cholinergic neurons (arrows) and axonal bundles (black with a magenta edge) are mutually exclusive. **D** Representative sample of a single coronal section of axonal bundle expression in the CPu measured approximately 0.50 mm from bregma. The section is slightly off the exact coronal plane as indicated by the presence of the lateral extent of the anterior commissure on the LH side and not the RH side. White regions delineate areas of axonal bundles. **E** Percent of axonal bundles is presented in relation to the total area of the CPu section (i.e. number of white pixels within the outline of the shape). **F** Heat map of axonal bundle density across the antero-posterior axis of the 59 sections investigated. The three-dimensional reconstruction of axonal bundles is presented in a collapsed two-dimensional plane. Density units identified the number of coronal sections where axonal bundles overlap. **G** Comparison of experimental observations and random simulations of the position of cholinergic neurons in the CPu. Simulations were conducted in the presence (red lines) and the absence (black lines) of axonal bundles. Abbreviations: RH: Right hemisphere, LH: Left hemisphere

To take account of the constraints that axonal fiber bundles impose on spatial properties, the volumes occupied by the myelinated fibers of passage were reconstructed by manual tracing throughout the CPu in registration with the cholinergic cell coordinates (Fig. 2A-C). These axonal bundle volumes were treated as exclusion zones in subsequent modelling of cell distributions for statistical analysis. Figure 2D shows the tissue that is left (in black) after subtracting the exclusion zones (white holes). The percentage of axonal bundles throughout the antero-posterior extent of the CPu was quantified (Fig. 2E). This area remained more or less constant throughout, with a mean of 26.81 ± 0.71% SD. The average axonal bundle density across the CPu was color-coded (Fig. 2F). These observations demonstrate that axonal fibers in the CPu comprise approximately ¼ of its total volume.

### Spatial Distribution of Cholinergic Neurons

Cell body distribution was evaluated by calculating Ripley’s K-functions for the experimentally observed spatial distribution and compared with (*n* = 1,000) randomly generated neuronal arrangements to determine whether there was any clustering. No normalization is needed when comparing regions, because the observed K-function is reported in comparison to the expected K-function, and computation of the expected K-functions takes account of the number and density of neurons in each region and the region’s volume and shape. In the CPu, this analysis demonstrated that there were statistically significant differences between the experimentally observed and homogeneous random distributions, suggesting clustering of cholinergic neurons. These differences were significant, *t*(99) = 9.10, *p* < 0.01, because of the large number of observations. However, the magnitude of these differences was small. To test whether the departure from randomness could be explained by the effects of the axonal bundles in the CPu, additional simulations were conducted in which neurons were excluded from the axonal bundle regions (Fig. 2G). These showed that there was indeed a significant effect of the axonal bundles, however, this could only account for a small proportion of the differences between the experimentally observed and simulated random distributions (Fig. 2G). The edge effects were not corrected because the comparison used a simulated distribution with the same edges, and the uncorrected measure of the simulated distribution was therefore similarly biased, as noted by (Baddeley et al., 1993).

**Fig. 3:**
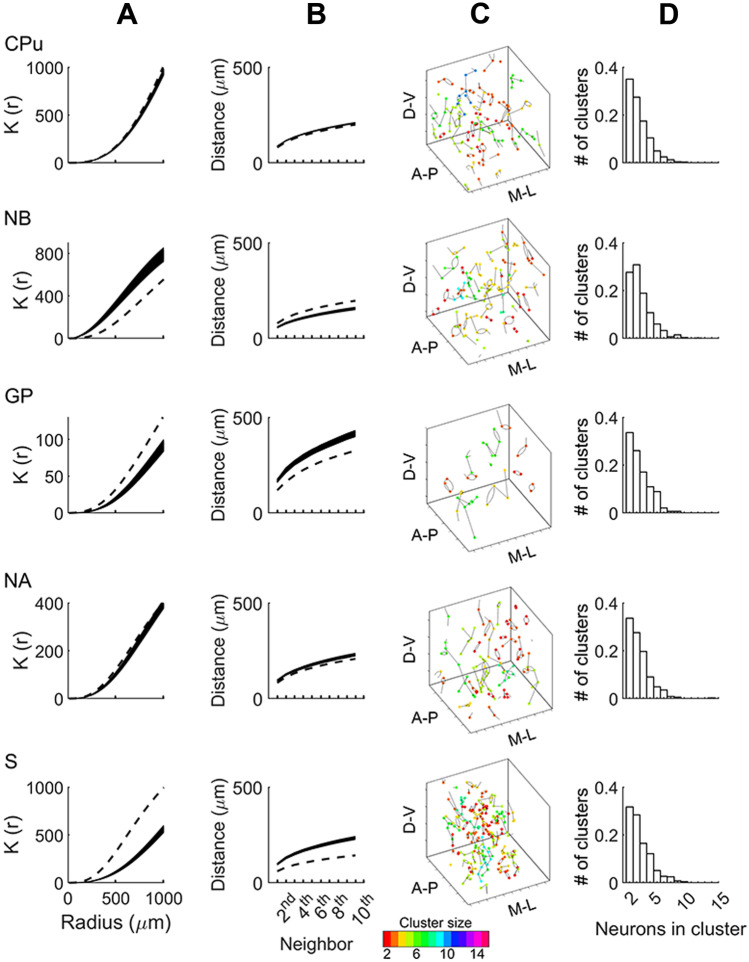
Spatial organization. Spatial organization of cholinergic neurons across the CPu, NB, GP, NA and the S. Ripley’s K-functions (column **A**), nearest neighbor distances (column **B**), representative samples of nearest neighbor clusters (column **C**), and nearest neighbor cluster group distributions (column **D**) are illustrated for each brain region examined. In columns A and B, K-functions and nearest neighbor distances distributions are compared between experimentally observed measures (dotted lines) and 1,000 simulations of homogeneous random distributions (solid lines). Abbreviations: CPu: caudate-putamen, NB: nucleus basalis, GP: globus pallidus, NA: nucleus accumbens and S: septum

Repetition of this analysis in the other regions studied, which did not have axonal bundles, showed that the GP, NA and S also showed an indication of clustering (Fig. 3A) that was statistically significant (GP: *t*(99) = 12.14, *p* < 0.001; NA: *t*(99) = 16.06, *p* < 0.001; and S: *t*(99) = 13.18, *p* < 0.001; simulated versus experimentally observed). These small *p* values arise because of the large *n* and do not indicate effect size (Sullivan & Feinn, 2012). For example, in the CPu the spatial difference between the simulated and experimental distribution at k = 80 is 10 µm, while in the GP at k = 80 the difference is 180 µm.

Considering the magnitude of the clustering, in the NA, as in the CPu, the magnitude was relatively small (for example, in NA, 100 cells lie within a radius of 460 µm, whereas in CPu 100 cells lie within a radius of 420 µm). In contrast, in the S the difference between simulated and experimentally observed distributions was relatively large (80 cells lie within a radius of 290 µm in the experimental and in 450 µm in the simulated). This was because the cholinergic cells in the experimentally observed distribution were mainly concentrated in the central part of the S, whereas in the simulation they were distributed throughout. On the other hand, in the NB, the difference between the experimentally observed and simulated Ripley’s K-function was in the opposite direction, indicating a spatial distribution with greater regularity than a random distribution *t*(99) = –17.5023, *p* < 0.01.

These observations were corroborated by measures of interneuron distance, which are inversely related to Ripley’s-K function. Analysis of nearest neighbor distances revealed differences across nuclei (S: 59.78 ± 0.86 µm; NB: 78.75 ± 0.86 µm; CPu: 80.84 ± 0.43 µm; NA: 82.14 ± 1.22 µm; GP: 118.54 ± 3.04 µm, mean ± SD) related in part to the differences in neural density in these regions. Comparison of interneuronal distance in random simulations (*n* = 1,000) and experimental observations across the 10 nearest neighbors of each cell indicated statistically significant clustering in CPu, S, NA and GP distributions but greater regularity in the spatial organization of cells in the NB (Fig. 3B). Together these analyses provide information about interneuronal distances in the cholinergic system and demonstrate the presence of distinct neuronal arrangements in subcortical nuclei.

Further analysis of the clustering of cells was based on concepts used in graph theory. A graph in this context is a mathematical structure used to represent pairwise relations between cells by lines between them (technically called edges). In Fig. 3C, the lines indicate the nearest neighbor of each cell. In graph theory a graph is called “connected” if there is a path via the edges from any node in the graph to any other. As shown in Fig. 3C, subsets of cells are connected by edges, which means that they are the nearest neighbors of each other. The number of cells in each connected subset is thus a measure of cluster size. We found that in each connected graph there was generally a progressive decrease in frequency with increasing cluster size. A slight exception to this trend was the NB, where the cluster with the greatest frequency had three cells rather than two cells, but after this peak the frequency also progressively decreased with increasing cluster size (Fig. 3D). Cluster size was not affected by addition of spatial noise to the coordinates (Supplementary Fig. 2). Overall, this analysis indicates that there are many small clusters of a few cells, but no large clusters of multiple cells in the subcortical regions studied.

**Fig. 4:**
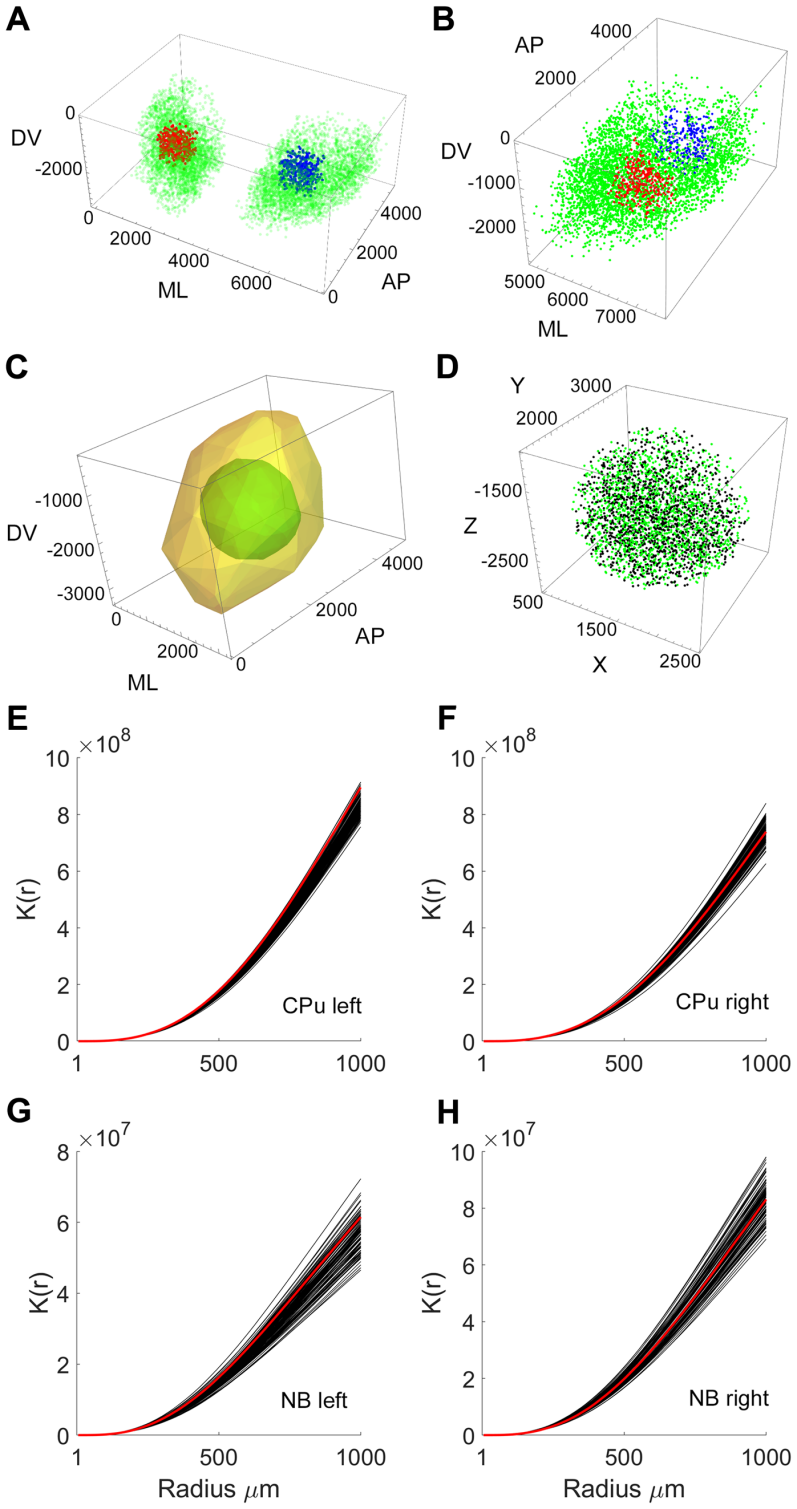
Analysis of inhomogeneous spatial distribution in CPu and NB. **A** Comparison of the density of neurons (green dots) of corresponding regions of left (blue dots) and right (red dots) CPu showed no significant difference. **B** Comparison of anterior (red dots) and posterior (blue dots) regions within CPu reveals inhomogeneity in density (see Table 2 for details). **C** Illustration of the region (sphere of radius 1 mm) extracted from the CPu to reduce edge effects on local density estimates. Local density was computed for the whole structure (yellow), and used to generate a spherical region (green) located some distance from the edges of the structure. **D** Example of the inhomogeneous Poisson distribution of points (green dots) generated from the local density estimates from the extracted region of the observed distribution (black dots). **E** – **H** Inhomogeneous Ripley’s K function for the observed (red line) and 99 simulated (black lines) spatial distributions from the CPu (Left and Right) and NB (Left and Right). Abbreviations: CPu: caudate-putamen; NB: nucleus basalis

The standard form of Ripley’s K function assumes homogeneity in the density of the spatial distribution. In the calculations of Ripley’s K function above, the comparison was made with a homogeneous, uniform distribution of constant density. However, previous work has indicated gradients in the density of cholinergic cells in the CPu (Bernacer et al., 2007; Hortnagl et al., 2020; Matamales et al., 2016) and NB (Hedrick & Waters, 2010). If density gradients exist and the distribution of cholinergic neurons is inhomogeneous then a different form of the K-function is required, K_inhom_(r) as defined in the Methods section. To test for homogeneity, we defined pairs of subregions in each structure and tested whether the point densities were equal. Comparing the density of cells in corresponding volumes on the left and right sides of the brain (Fig. 4A, Table 2) revealed no significant side difference in density in the areas tested. However, we found significant departure from homogeneity within both the CPu and NB (Fig. 4B, Table 2), which may have caused the difference between the simulated and observed distribution in these regions.

**Table 1:** Characteristics of cholinergic neurons, and structures, across hemispheres in the mouse forebrain. Abbreviations: GP: globus pallidus, NA: nucleus accumbens, S: septum, NB: nucleus basalis, CPu: caudate-putamen

*Total/absolute number*					
	**GP**	**NA**	**S**	**NB**	**CPu**
**Left hemisphere**	308	638	638	1130	4002
**Right hemisphere**	200	619	827	1078	4549
*Total (absolute) volume of structures*					
	**GP**	**NA**	**S**	**NB**	**CPu**
**Left hemisphere**	2.74 mm^3^	2.13 mm^3^	0.89 mm^3^	3.37 mm^3^	15.06 mm^3^
**Right hemisphere**	2.07 mm^3^	1.78 mm^3^	1.17mm^3^	2.95 mm^3^	15.95 mm^3^
*Density of cholinergic neurons*					
	**GP**	**NA**	**S**	**NB**	**CPu**
**Left hemisphere**	112.41 mm^−3^	299.53 mm^−3^	716.85 mm^−3^	335.31 mm^−3^	265.74 mm^−3^
**Right hemisphere**	96.62 mm^−3^	347.75 mm^−3^	706.84 mm^−3^	365.42 mm^−3^	285.20 mm^−3^

**Table 2:** Spatial analysis – Statistical test for inhomogeneity. Abbreviations: GP: globus pallidus, NA: nucleus accumbens, CPu: caudate-putamen, NB: nucleus basalis, S: septum

	**Anterior vs Posterior (Left)**	**Anterior vs Posterior (Right)**	**Left vs Right**
GP	F = 1.18 df = [103,87] *p* = 0.21	F = 1.31 df = [89,117] *p* = 0.08	F = 1.09 df = [117,127] *p* = 0.32
NA	F = 1.05 df = [437,415] *p* = 0.30	F = 1.03 df = [345,357] *p* = 0.37	F = 1.10 df = [397,437} *p* = 0.16
CPu	F = 2.10 df = [1119,533] *p* = 0.0 × 10^–9^	F = 1.51 df = [479,725] *p* = 2.39735 × 10^–7^	F = 1.09 df = [725,667] *p* = 0.14
NB	F = 1.38 df = [265,365] *p* = 0.0023	F = 2.43 df = [95,231] *p* = 2.9208 × 10^–8^	F = 1.01 df = [631,627] *p* = 0.47
S	F = 1.05 df = {307,293] *p* = 0.34	NA	NA

To take account of density gradients within the CPu and NB, we used an extended version of Ripley’s *K* function for non-constant intensity spatial point processes (Baddeley et al., 2001). The inhomogeneous *K* function was estimated by summing the reciprocal of the product of local densities for all pairs of points separated by a distance less than *r* (Marcon & Puech, 2009). The confinement of cholinergic neurons within specific nuclei induces a systematic negative bias in intensity estimates at the boundary of the nucleus (Burguet & Andrey, 2020). To overcome this effect in the CPu we extracted a sub-region (Fig. 4C, D) that was separated from the boundary and estimated the intensity from the total region. Using this approach, the effect of the boundary on the density measure was reduced. To determine the local densities we used a kernel estimation (Silverman, 1986) which smoothed the local fluctuations. Following the standard Monte Carlo approach (Baddeley et al., 2014), we compared the observed and simulated ensemble using K_inhom_(r). As before, edge correction was not undertaken on the basis that observed and simulated distributions had the same 3D shape and the K-function would therefore be similarly biased (Baddeley et al., 1993, 2014). The inhomogeneous K-function analysis revealed that the spatial distribution of cholinergic cells in the CPu and NB (both left and right sides) was not significantly different from that predicted by the null hypothesis of inhomogeneous Poisson distribution with similar density gradients to the observed distribution (Fig. 4E–H). Thus the clustering in the CPu and NB could be explained by the gradient in density.

### Total Regional Volumes and Inter-neuron Volumetric Properties

**Fig. 5:**
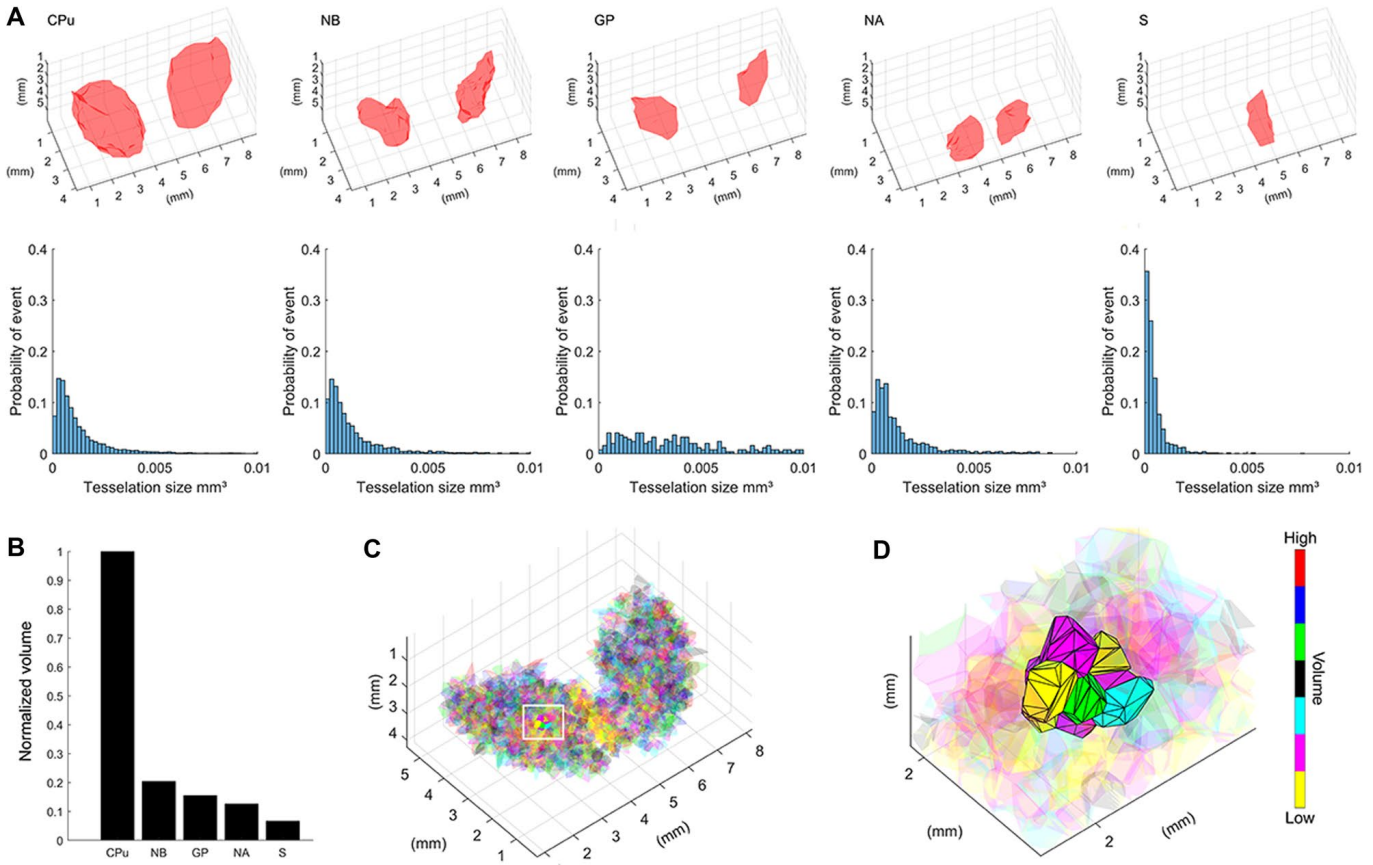
Volumetric properties of the brain regions examined. **A** Three-dimensional reconstruction of brain regions examined (top row, left structures illustrate the right hemisphere) and frequency distribution functions of inter-neuronal volume for each region investigated (right and left hemisphere data are combined for each structure examined, bottom row). **B** Normalized histogram of the bilateral volume of each brain region examined. **C** Three-dimensional Voronoi tessellations calculated from centroid locations of cholinergic neurons across the five subcortical regions examined. Color codes in Voronoi tessellations identify volume size. **D** Close-up view of Voronoi tessellations within a CPu sector (white rectangle in panel ***C***). Abbreviations: CPu: caudate-putamen; NB: nucleus basalis GP: globus pallidus, NA: nucleus accumbens and S: septum

Reconstructions of area boundaries in the set of serial sections was used to determine volumetric measures across subcortical regions. The largest nucleus measured was the bilateral CPu (31.01 mm^3^) followed by the NB (6.32 mm^3^), GP (4.81 mm^3^), NA (3.91 mm^3^) and S (2.06 mm^3^) (Fig. 5A, B). The availability of space for individual cholinergic neurons to innervate and receive inputs from other cells is a determinant of connectivity. The three-dimensional spatial distribution of the cholinergic neurons regulates the space around each neuron. The available volume around each cell body can be quantified by Voronoi tesselations (Fig. 5C, D). Within each region, Voronoi tessellations were generated using the coordinates of the centroid of the cell body of each cholinergic interneuron. Mean Voronoi volumes for neurons in the CPu, NB, and NA were similar to each other (CPu: 0.002 ± 0.00003 mm^3^, NB: 0.0017 ± 0.00006 mm^3^, NA: 0.0015 ± 0.00008 mm^3^, mean ± SD), but different from the smaller volumes in the S (0.0005 ± 0.00002 mm^3^) and the larger volumes in the GP (0.0053 ± 0.0004 mm^3^). The smaller volumes in the S may reflect the clustering of neurons toward the center of the nucleus. The larger volumes in the GP may reflect the relative amount of white matter compared to other brain regions investigated. Interneuronal space distributions are presented in Fig. 5A bottom row.

## Discussion

The present study examined spatial distributions of cholinergic cell bodies and axonal bundles of subcortical nuclei implicated in cognitive modulation. Topological analyses identified three structural principles in neuronal architectures. Approximately 27 percent of the CPu is composed of axonal bundles from which cell bodies are excluded, constraining the location of cells in this region. Analysis of spatial distribution using Ripley’s K-function revealed statistically significant differences between subcortical brain regions containing cholinergic neurons. Compared to a homogeneous uniform distribution, the GP, NA, CPu, and S showed indications of clustering of cell bodies and, in contrast, the NB was more regular. These findings were supported by nearest-neighbor measures of interneuron distance. However, the clustering in the CPu and regularity in the NB could be explained by the inhomogeneity due to gradients in the density. Analysis of clusters of neurons forming connected subsets revealed that cluster sizes showed a progressive decrease in frequency from 2 to 14 cells in the CPu, S, NA and GP, whereas in NB the maximum cluster frequency size was three cells. Consistent with these results, volumetric analysis showed similarities in interneuronal volumes across subcortical nuclei with smaller volumes dominating. Third, the number of cholinergic neurons varies as a function of the volume of a specific brain region but neuronal volumes are constant between regions. These observations provide a topographic description of cholinergic neurons and demonstrate spatial differences in cognitive control networks. This information is important for future digital cellular atlases and computational models of the forebrain cholinergic system enabling models based on actual spatial geometry (Erö et al., 2018).

### Methodological Considerations

The findings of the present study are based on an analysis of 14,000 neurons from a single female animal. This exhaustive approach was used because the research question necessitated a full analysis of the large data set created by high resolution imaging over the entirety of each brain region. The imaging provided accurate 3-D location in a coordinate system allowing analysis of all interneuronal distances. Analysis of the images and calculation of the measures used required considerable computational resources. This limited to one, the number of animals that could be analyzed in this way. The strategy of using a single animal when such data sets are required has been applied in previous studies (Schneider et al., 2019). Neuroanatomical studies of sex differences in transgenic mouse models have reported that female mice have approximately 34% less basal forebrain cholinergic neurons compared with genotype-matched males (Kelley et al., 2014). Future experiments should investigate differences in the spatial organization of cholinergic neurons between female and male mice.

The transgenic mouse line used in the present study may have differed anatomically from wild type mice as evident in the measure of the CPu antero-posterior distance (present study: 3.54 mm; atlas of Franklin and Paxinos (2008): 4.5 mm). Despite this variation, the measurements of average somal size in the current study are consistent with measurements made in wild-type mice (Hedrick & Waters, 2010). Thus although the present study is based on analysis of a transgenic mouse brain, the relative spatial measures of cholinergic neurons are expected to be representative of the mouse type.

Data collection techniques used in the present investigation were selected to maximize image resolution (X: 0.425 μm, Y: 0.425 μm, Z: 1.4 μm) and improve measures of cell body volume. This approach resulted in big-data sets (> 50 terabytes) that demanded parallel computing approaches to measure neuronal and axonal contours. The approach required the reconstruction of neuronal maps in the antero-posterior axis by following anatomical landmarks of the mouse forebrain. The overlapping of 60 μm sections by computer algorithms and human supervision generated a three-dimensional map. Employing this technique provided high accuracy in the medio-lateral and dorso-ventral direction. Successive sections could be accurately aligned in those directions. There may be small errors in the antero-posterior measurements due to the planar orientation of each section, but these are not cumulative. Ripley’s K-function analyses revealed clustered and regular distributions of cholinergic cell bodies. While these observations were statistically significant, proximity between data points and simulation functions in the CPu and NA, and in the NB taking account of inhomogeneity, indicate that clustering is not constrained to specific radii and approaches randomness. This information will be important in the development of striatal models of cognitive modulation (Hjorth et al., 2020).

A common source of error in three-dimensional spatial analysis is that section to section alignment may vary by µm amounts due to unavoidable variability in mounting. However, this error of a few µm is unlikely to affect the analysis because of the low density of cholinergic neurons and the interneuronal distances on the order of 60–120 µm. The inevitable shrinkage that occurs during tissue preparation – provided it is uniform—is not expected to affect the conclusions of the K-function analysis, because the analysis is based on a comparison of observed and simulated distributions which have the same total number and volume. Similarly, although the absolute value of the nearest-neighbour distance is affected by shrinkage, the value for the observed distribution is always compared to a simulated distribution with the same total number and volume. The successful alignment of medial and lateral boundaries from section to section indicated that shrinkage was uniform between sections. Differential shrinkage between areas remains a possibility, and hence comparisons of absolute values between areas should be made cautiously.

Despite efforts to acquire the locations of the entire population of cholinergic neurons in the regions of interest, half of one tissue-section (the left hemisphere part of the 6^th^ section from the anterior pole of the CPu, out of a total 59 sections) was damaged and could not be included in the analysis of the dataset. This suggests that less than 1% of cells were missing from the sample, affecting only the CPu and NA. However, simulation studies have shown that the K-function is relatively robust against missing data. For example, missing data did not cause significant effects on the estimation of the K-function when 10% of the data was missing in analyses described by Arbia et al. (2017). Therefore, it is unlikely that the less than 1% of data missing in the present study would alter the results of the analysis.

### Cell Counts

The purpose of the paper was to provide a database for analysis of the 3D spatial distribution of forebrain cholinergic neurons, rather than estimates of total numbers. Other approaches, such as stereology, are more suitable for estimating absolute total numbers in each structure, because the use of sampling methodology allows more detailed examination of individual neurons across a number of different animals. To date there have been no reports of studies specifically designed to determine the total number of cholinergic interneurons in the normal mouse CPu. However, as part of another study, Peterson et al., (1999) reported a total number of 4,704 ± 509 ChAT-positive neurons in the CPu unilaterally, which if symmetrical would give an extrapolated total range of 8,390 – 10,426. Similarly, Deng and Reiner (2016) counted ChAT-positive neurons in the anterior 2/3 of the dorsal CPu (from the rostral tip of the CPu to the anterior commissure) and reported a total ranging from 2,730 to 3,300 cholinergic interneurons unilaterally (in their Fig. 1, Deng & Reiner, 2016). Extrapolating to the whole CPu bilaterally (assuming symmetry) gives a range of 8,190 – 9,900 cholinergic interneurons in total. Thus, the total number of CPu cholinergic interneurons that we report (8,551) is within the range of total numbers published using explicit stereological counting methods.

If we knew the proportion of all mouse CPu neurons that are cholinergic and the total number of all neurons, we could use this to obtain another estimate of the total number of CPu cholinergic interneurons. The often quoted proportion for rat and primate brains is 1 – 3% (Mallet et al., 2019). This compares with stereological studies indicating 1% in the monkey (Deng et al., 2010) and 0.4% in the rat (Oorschot, 2013). In the mouse, the best available estimate of the proportion of all CPu neurons that are cholinergic comes from Peterson et al. (1999) whose data indicate a range of 0.25% – 0.31%. Data on the total number of all neurons in the mouse CPu is available, indicating a total number of all types of neurons of 1.5 – 1.7 million cells unilaterally (Hickey et al., 2008; Peterson et al., 1999; Rosen & Williams, 2001; Slow et al., 2003). Therefore, based on the proportion of cells that are cholinergic and the total number of all neurons, we expect a range of 7,500 – 10,540 cholinergic interneurons bilaterally in the mouse. Thus, the number of detected neurons in the CPu in our study is within the range expected.

### Cell Volumes

Measures of cell body diameter in the present investigation are consistent with previous reports of cholinergic cell topology (Nakajima et al., 1985; Wu et al., 2014; Zhou et al., 2002). The mean diameter of the imaged cells was 25.37 μm and did not significantly vary across subcortical nuclei. In contrast, electrophysiological investigations have revealed differences in firing rate features across cholinergic neurons. For example, it has been reported that cholinergic neurons in the CPu (2–10 Hz) and NA (0.6—12 Hz) display tonic activity (Carrasco et al., 2020; Pappas et al., 2018; Wilson et al., 1990), but in the NB cholinergic neurons remain silent at rest and have spontaneous irregular spikes (3 Hz) (Hedrick & Waters, 2010). Therefore, firing characteristics of cholinergic neurons do not appear to be determined by somatic size. This observation is supported by computer models that have demonstrated that neuronal response properties (tonic vs. phasic) are regulated by dendritic topology (van Elburg & van Ooyen, 2010). On the other hand, a common feature of forebrain cholinergic projection neurons and interneurons is an extremely large and extensive axonal arborization. In the mouse, the axons of forebrain cholinergic projection neurons project widely (Kozlowski et al., 2020) and are estimated to produce 50 cm of axon per neuron (Wu et al., 2014). Comparative data for striatal cholinergic interneurons is not available, but they are also reported to be very extensive and to have about 500,000 cholinergic varicosities per neuron (Contant et al., 1996; Descarries & Mechawar, 2000; Zhou et al., 2002). Thus, it is possible that the similar, large somal size reflects the metabolic demands of synthesis and release of acetylcholine from numerous release sites distributed over an extensive axonal arborization, both in interneurons and projection neurons.

### Network Topology

Previous work by Matamales et al. (2016) focusing on the CPu showed a minor degree of clustering of cholinergic interneurons but a predominantly random organization. The present results extend these findings by showing that the fascicles in which bundles of axons traverse the CPu had only a minor effect on the spatial organization, but on the other hand, when the inhomogeneity in density was taken into account it was found that the clustering could be explained by the density gradient. Examination of the clustering in other nuclei also revealed that, in contrast, NB neurons are regularly distributed compared to a uniform random distribution, or randomly distributed when compared to an inhomogeneous Poisson distribution. A plausible explanation for the observed variations in neuronal organization is topographic restrictions imposed by afferent neurons (Liu et al., 2015; Selemon & Goldman-Rakic, 1985). The nature of the projections from NB, GP and S cholinergic neurons (Chaves-Coira et al., 2018; Javed & Cascella, 2020; Khakpai et al., 2013; Nemy et al., 2020) may also impose different constraints than those caused by the intrinsic microcircuits in the CPu (Hjorth et al., 2020) and NA (Salgado & Kaplitt, 2015). Mapping the synaptic afferents to and efferents from single cholinergic neurons in the examined brain regions will be needed to validate this proposal and further extend the model of spatial architecture identified in the present investigation.

The present work complements a previous analysis of clustering of the basal forebrain cholinergic projection neurons (Nadasdy et al., 2010) by adding a comparison with cholinergic interneurons in the CPu and NA and formal statistical spatial analysis. The definition of clustering used in Nadasdy et al., (2010) is different from the definition we are using, which is based on Ripley’s classical paper (Ripley, 1977). Specifically, (Nadasdy et al., 2010) visualized inhomogeneous regions of locally higher density which is different from statistically determining whether clustering is occurring in excess of that predicted by the regional density. We undertook statistical analysis of the spatial distribution using tools for spatial analysis that have been developed by statisticians for spatial analysis in diverse fields. These tools are increasingly used to rigorously determine whether clustering is statistically significant and the Ripley K-function (Ripley, 1977), in particular, has been extensively used and studied. These analyses are also used in neuroscience (Jafari-Mamaghani et al., 2010; Matamales et al., 2016; Rafati et al., 2016) and in this paper we have extended the analysis to three dimensions and (to the best of our knowledge) for the first time, applied inhomogeneous K-functions (Baddeley et al., 2001; Marcon & Puech, 2009) to cell distributions. Using this methodology is helpful in distinguishing between whether clustering is statistically real or due to inhomogeneity in the density of the cells of interest (Baddeley et al., 2001; Christensen & Møller, 2020). As evidenced in our results, this a biologically important consideration.

## Information Sharing Statement

The database of coordinates that supports the findings of this study is available as an electronic file in the supplementary materials.

## Supplementary Information

Below is the link to the electronic supplementary material.

Supplementary file1 (MP4 25496 KB)

Supplementary file2 (XLSX 611 KB)

Supplementary file3 (DOCX 2.57 MB)

## Data Availability

Not applicable.
