# Supplementary material for: Three-Dimensional Spatial Analyses of Cholinergic Neuronal Distributions Across The Mouse Septum, Nucleus Basalis, Globus Pallidus, Nucleus Accumbens, and Caudate-Putamen
Source: Neuroinformatics. 2022 Jul 6;20(4):1121–36. doi: 10.1007/s12021-022-09588-1 (PMC9588480; doi:10.1007/s12021-022-09588-1)
Supplement: Supplementary file 3 — Supplementary file3 (DOCX 2.57 MB) [file 12021_2022_9588_MOESM3_ESM.docx]

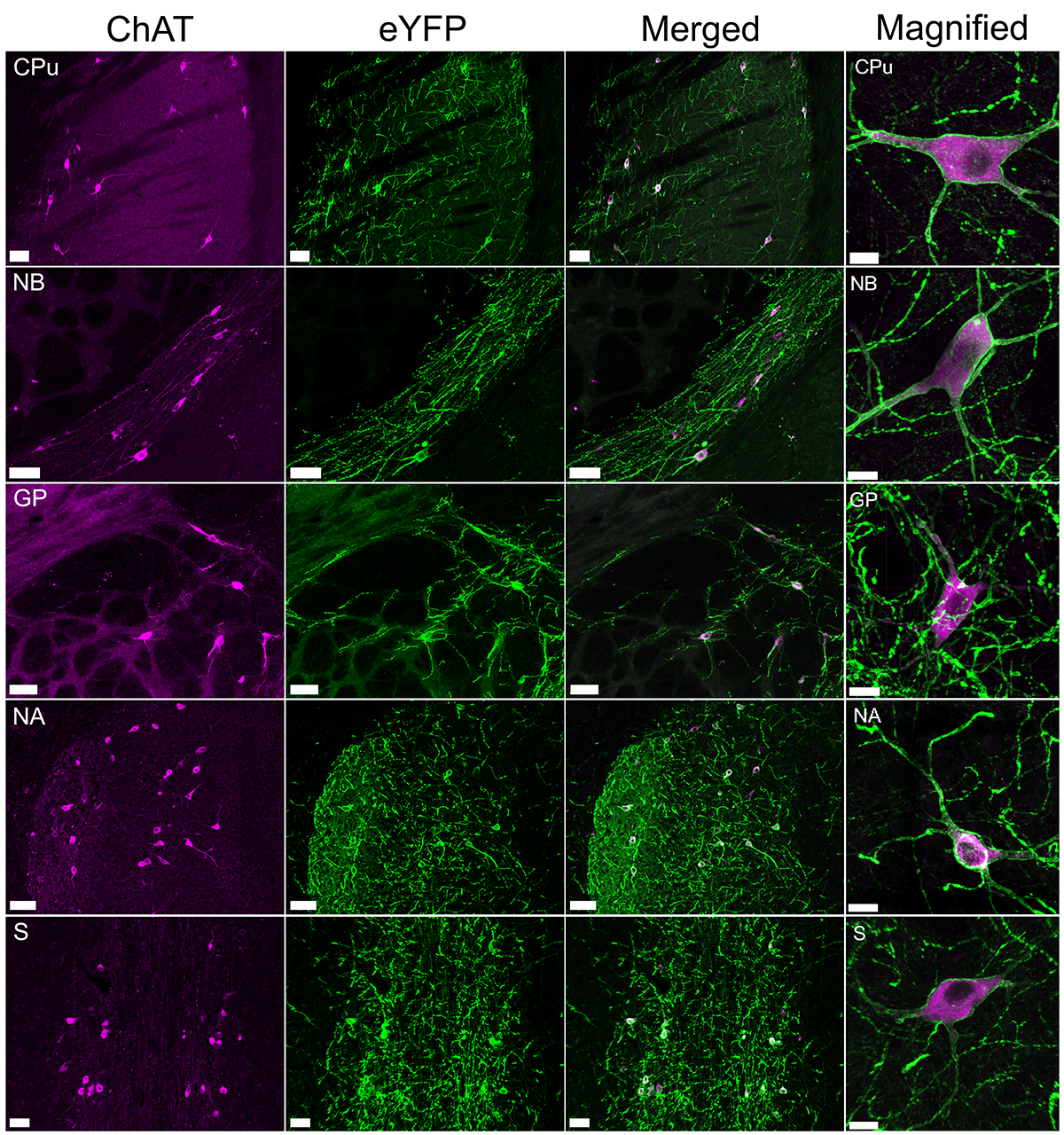


**Supplementary Figure 1.**

Identification of cholinergic neurons.

Representative expression of ChAT (magenta) and eYFP (green) signals in the striatum. Co-localization of signals is presented in the ‘merged column’. Scale bar 50 μm. (first three columns). Representative high magnification microphotographs of cholinergic neurons from each brain region examined are presented in the far right column. Scale bars are 10 μm.

Abbreviations: CPu: caudate-putamen; NB: nucleus basalis GP: globus pallidus, NA: nucleus accumbens and S: septum.


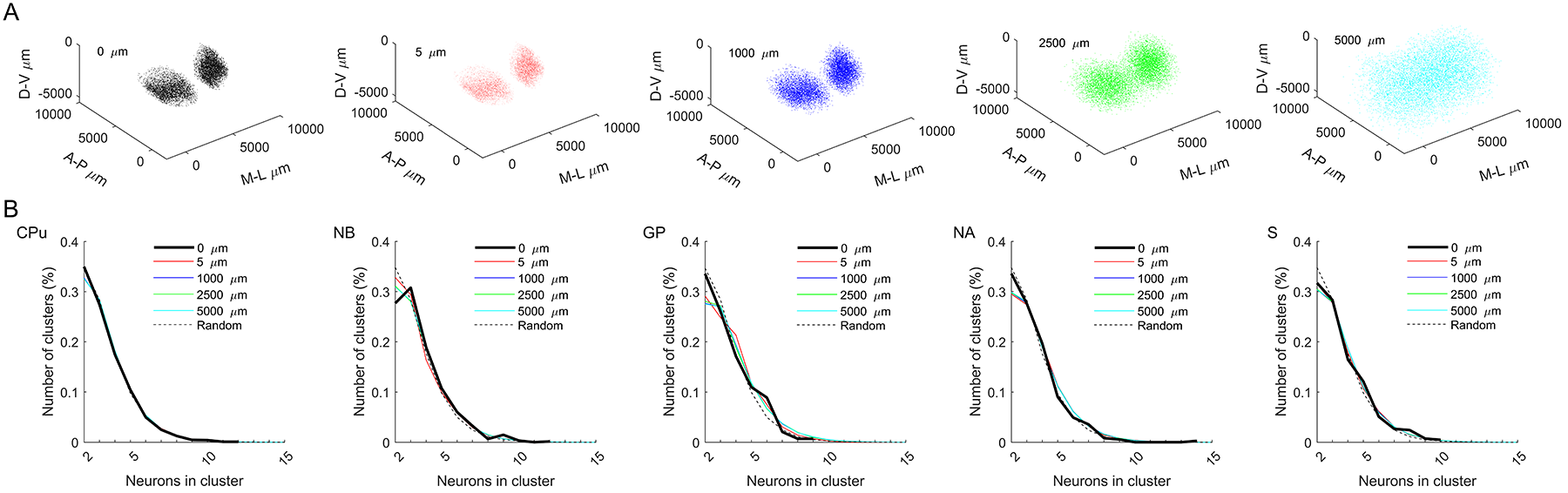


**Supplementary Figure 2.**

Effects of spatial noise on cluster size.

1. Using the example of the CPu, five different amounts of noise was added to the x, y, z coordinates. From left to right, 0, 5, 1000, 2500, 5000 µm.
2. Nearest neighbor cluster group distributions with various levels of spatial noise.

Abbreviations: CPu: caudate-putamen; NB: nucleus basalis GP: globus pallidus, NA: nucleus accumbens and S: septum.
